# The Neurite Outgrowth Inhibitory Nogo-A-Δ20 Region Is an Intrinsically Disordered Segment Harbouring Three Stretches with Helical Propensity

**DOI:** 10.1371/journal.pone.0161813

**Published:** 2016-09-09

**Authors:** Viviane Zelenay, Michael E. Arzt, Stefan Bibow, Martin E. Schwab, Roland Riek

**Affiliations:** 1 Department of Physical Chemistry, ETH Zurich, Zurich, Switzerland; 2 Brain Research Institute, University of Zurich, Zurich, Switzerland; 3 Department of Biology, ETH Zurich, Zurich, Switzerland; 4 Department of Health Sciences and Technology, ETH Zurich, Zurich, Switzerland; George Washington University, UNITED STATES

## Abstract

Functional recovery from central neurotrauma, such as spinal cord injury, is limited by myelin-associated inhibitory proteins. The most prominent example, Nogo-A, imposes an inhibitory cue for nerve fibre growth via two independent domains: Nogo-A-Δ20 (residues 544–725 of the rat Nogo-A sequence) and Nogo-66 (residues 1026–1091). Inhibitory signalling from these domains causes a collapse of the neuronal growth cone via individual receptor complexes, centred around sphingosine 1-phosphate receptor 2 (S1PR2) for Nogo-A-Δ20 and Nogo receptor 1 (NgR1) for Nogo-66. Whereas the helical conformation of Nogo-66 has been studied extensively, only little structural information is available for the Nogo-A-Δ20 region. We used nuclear magnetic resonance (NMR) spectroscopy to assess potential residual structural propensities of the intrinsically disordered Nogo-A-Δ20. Using triple resonance experiments, we were able to assign 94% of the non-proline backbone residues. While secondary structure analysis and relaxation measurements highlighted the intrinsically disordered character of Nogo-A-Δ20, three stretches comprising residues ^561^EAIQESL^567^, ^639^EAMNVALKALGT^650^, and ^693^SNYSEIAK^700^ form transient α-helical structures. Interestingly, ^561^EAIQESL^567^ is situated directly adjacent to one of the most conserved regions of Nogo-A-Δ20 that contains a binding motif for β1-integrin. Likewise, ^639^EAMNVALKALGT^650^ partially overlaps with the epitope recognized by 11C7, a Nogo-A-neutralizing antibody that promotes functional recovery from spinal cord injury. Diffusion measurements by pulse-field gradient NMR spectroscopy suggest concentration- and oxidation state-dependent dimerisation of Nogo-A-Δ20. Surprisingly, NMR and isothermal titration calorimetry (ITC) data could not validate previously shown binding of extracellular loops of S1PR2 to Nogo-A-Δ20.

## Introduction

Neurons in the central nervous system (CNS) exhibit very limited capacity to regrow upon neurotrauma, preventing them from restoring disrupted networks after a spinal cord or brain injury. This is contrary to the situation in the peripheral nervous system (PNS), where regrowth of nerve fibres can occur to a much higher extent [1, 2]. CNS-specific myelin-associated inhibitory molecules that actively prevent the outgrowth of neurons are an important factor accounting for this discrepancy [3].

One of the most prominent members of these inhibitory molecules is the 1163 residues (rat sequence) long membrane protein Nogo-A, also referred to as reticulon 4-A [4–6]. Nogo-A is expressed on the surface of oligodendrocytes where it exhibits an inhibitory signal for neurite growth [7, 8]. Nogo-A acts as a stabilizer for the highly complex CNS wiring; it restricts synaptic plasticity and influences various intracellular processes such as shaping of the endoplasmic reticulum (ER), where particularly high Nogo-A levels are found [9–11]. Two domains of Nogo-A have been identified that impose inhibitory effects on neurite growth and cell migration: Nogo-A-Δ20 and Nogo-66 [7]. The Nogo-A-Δ20 domain, which contains 182 residues, is located in the middle of the 803 residues long Nogo-A-specific segment. In contrast, the 66 residues long Nogo-66 domain is situated between two long hydrophobic stretches at the C-terminus that Nogo-A shares with its much smaller isoforms Nogo-B and Nogo-C, as well as with other reticulon proteins. Neurons express distinct receptors for each of these inhibitory domains, i.e., sphingosine 1-phosphate receptor 2 (S1PR2) together with tetraspanin-3 for Nogo-A-Δ20 and Nogo receptor 1 (NgR1) in association with co-receptors p75, Troy and Lingo-1 for Nogo-66 [12–18]. S1PR2 and NgR receptor complexes both lead to an activation of RhoA in the neuronal cytoplasm, which in turn causes destabilisation of the actin cytoskeleton and thus collapse of the neuronal growth cone as well as a general downregulation of the neuronal growth machinery [10].

Structural analysis at atomic resolution is a powerful approach to gain insight into the structure-activity relationship of proteins. To date, Nogo-66 is the only inhibitory domain of Nogo for which a structure has been determined [19]. For Nogo-A-Δ20, it is only known that it exhibits an overall unstructured conformation [20, 21]. However, according to circular dichroism (CD) spectroscopy, this region seems to contain residual secondary structure [21]. This is supported by secondary structure prediction indicating the presence of residual conformations within the Nogo-A-Δ20 sequence [20, 22]. Furthermore, the addition of zinc ions to Nogo-A-Δ20 induced a higher degree of α-helical content in circular dichroism [21]. However, the exact locations of putative structural elements within Nogo-A-Δ20 have not been known until now.

The interaction between Nogo-A-Δ20 and the G-protein coupled receptor S1PR2 has been demonstrated biochemically and functionally [12]. Extracellular loops (ECLs) 2 and 3 of S1PR2 were concluded to be the primary binding sites for Nogo-A-Δ20, based on the nanomolar affinities of isolated ECL peptides in a microscale thermophoresis assay. However, only little is known about the exact binding mode and amino acid residues involved in this interaction.

Here, we provide first structural data of Nogo-A-Δ20 with single-residue resolution using nuclear magnetic resonance (NMR) spectroscopy. The backbone of biologically active Nogo-A-Δ20 was assigned to a completeness of 94% using various triple resonance experiments, revealing three sites of marked α-helical propensity. A concentration-dependent dimerisation was found using diffusion NMR experiments. In addition, we investigated the interaction of S1PR2 with Nogo-A-Δ20 by titrating isolated ECL2 and ECL3 of S1PR2 to Nogo-A-Δ20. No conclusive binding data were obtained for these isolated fragments using NMR spectroscopy and isothermal calorimetry (ITC).

## Results

### Structural Propensities for Nogo-A-Δ20

**Fig 1:**
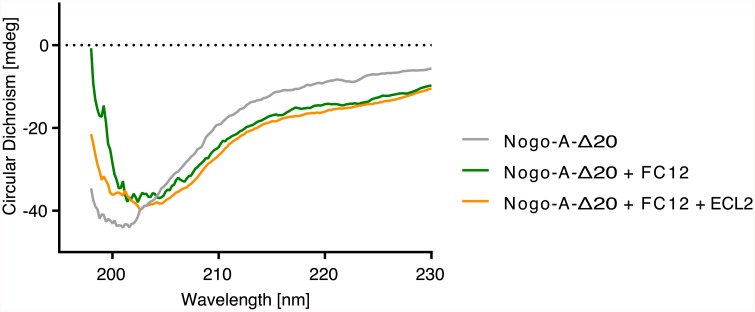
CD spectroscopy of Nogo-A-Δ20 at 25°C. Recombinant Nogo-A-Δ20 exhibits a spectrum typical for unstructured proteins. Addition of FC12, thought to mimic a membrane environment, slightly enhances the structural composition of the protein. However, the observed changes are negligible compared to the α-helical structure reported for Nogo-66 upon FC12 addition [19]. Addition of ECL2 to Nogo-A-Δ20 in a membrane-mimicking environment does not lead to folding, either.

Nogo-A-Δ20 was expressed as ^13^C- and/or ^15^N-labelled recombinant protein in *E*. *coli* to study its structural characteristics using CD and NMR spectroscopy. The CD spectrum of Nogo-A-Δ20 with its minimum at around 200 nm suggests a high proportion of unstructured regions, with some residual secondary structure (Fig 1). Addition of dodecylphosphocholine (FC12), which is required for structuring of Nogo-66 [19], only led to minor changes in the CD spectrum of Nogo-A-Δ20, indicating that FC12 induced no significant structural rearrangements.

**Fig 2:**
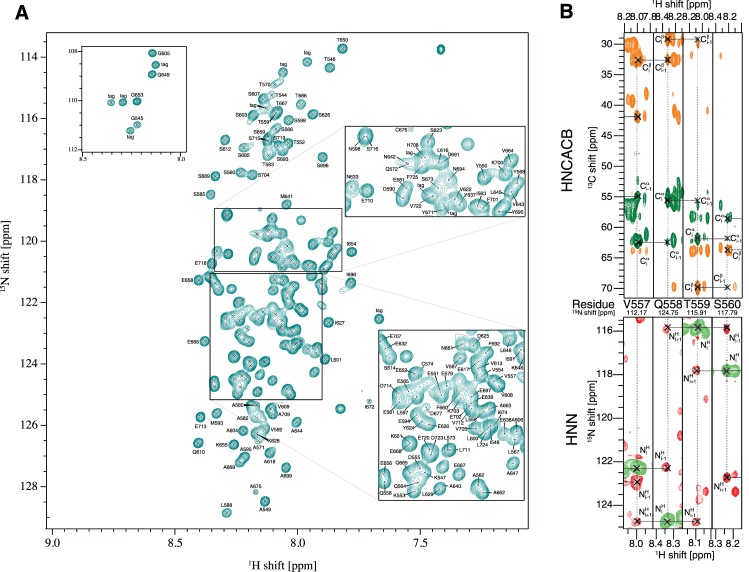
2D [^15^N, ^1^H]-HSQC and some strips of 3D triple resonance experiments used for the sequential assignment of Nogo-A-Δ20. **A**: Assigned [^15^N, ^1^H]-HSQC of Nogo-A-Δ20 following the numbering scheme of rat Nogo-A. The narrow chemical shift dispersion is a common feature of intrinsically disordered polypeptides (IDPs). **B**: At the top, strips of the 3D HNCACB spectrum are shown with green and orange contours indicating positive and negative cross peaks, respectively. At the bottom, strips of the 3D HNN spectrum are shown with red and green contours indicating positive and negative cross peaks, respectively. In the HNCACB, cross peaks belonging to C^α^, C^α^_i-1_, C^β^, and C^β^_i-1_ are indicated, while in the HNN spectrum, the N_i_, N_i-1_, and N_i+1_ are labelled. The HNCACB spectrum was recorded at a 600 MHz and the HNN spectrum was recorded at a 700 MHz NMR spectrometer at 6°C and pH 7.4.

In accordance with the CD spectrum observed for Nogo-A-Δ20, a 2D-NMR [^15^N,^1^H]-HSQC spectrum confirmed the intrinsically disordered character of Nogo-A-Δ20, as deduced from the low chemical shift dispersion in the ^1^H dimension (Fig 2A). In order to obtain sequence-specific conformational and structural information, a backbone assignment was conducted. Standard pulse programs (HNCA, HNCACB) and non-standard experiments (HNN and HCAN) were recorded on [^13^C, ^15^N]-Nogo-A-Δ20. The low dispersion in the proton dimension, present in HNCA, HNCACB, and HNN spectra, together with many proline residues present in the sequence (13%, 23 prolines of 182 residues), interrupted the sequential assignment and posed a severe challenge. To overcome the discontinuity of the spectra along the backbone caused by proline residues, an HCAN spectrum was recorded. Here, the magnetisation is transferred from ^1^H^α^ to ^13^C^α^ and further on to N_i_ and N_i+1_, enabling a connection of a proline to its following residue [23] and allowing a sequential assignment through prolines. With this set of NMR experiments, 94% of the non-proline ^13^C^α^ and ^13^C^β^ and 83% of proline ^13^C^α^- and ^13^C^β^-frequencies in Nogo-A-Δ20 were assigned (Fig 2B). An unambiguous assignment was impossible for the residue stretches ^575^PSFE^578^ and ^678^LIKETK^683^ due to severe peak overlap (S1 Fig). The complete assignment can be found in Fig 2A and was deposited in the Biological Magnetic Resonance Bank (BMRB) with the ID 26653.

**Fig 3:**
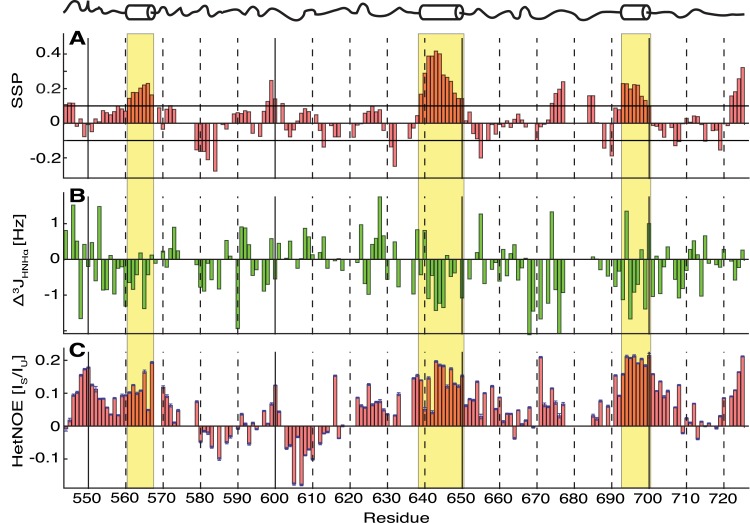
Secondary structure and flexibility analysis of Nogo-A-Δ20. **A**: Secondary structure propensities for single residues. Three residual α-helices were found as defined by five or more consecutive residues with SSP values above 0.1 (yellow boxes). **B**: Δ^3^J_HNHα_ derived from the difference between ^3^J_HNHα_ measured from intensity modulated [^15^N,^1^H]-HSQC experiments and corresponding random coil values [29]. Negative Δ^3^J_HNHα_ indicate an α-helical while positive values indicate an extended conformation, respectively. **C**: ^15^N{^1^H}-HetNOEs. A value near 1 indicates a fully rigid conformation, values close to zero indicate the presence of ~1 ns dynamics, while negative values indicate the presence of even faster motion. Most of the values are positive between the ratios 0.1–0.2. Only two consecutive strands between the residues 580–588 and 603–614 have negative values. Error bars were calculated using Gaussian error propagation. The locations and secondary structure propensity of α-helices as determined by NMR (see text) are indicated above the diagrams. The cylinders/yellow boxes indicate transient α-helical propensities. Residues belonging to the affinity tags flanking Nogo-A-Δ20 are not plotted.

The sequential assignment enables secondary structure analysis using secondary chemical shifts of Δδ^13^C^α^ and Δδ^13^C^β^, which are the difference between the observed chemical shifts and corresponding random coil chemical shifts (S2 Fig) [24]. Positive Δδ^13^C^α^ values and negative Δδ^13^C^β^ values for several consecutive residues indicate an α-helical conformation. Conversely, negative Δδ^13^C^α^ in combination with positive Δδ^13^C^β^ indicate the formation of a β-strand. These two experimental values can be combined with the secondary structure propensity (SSP) algorithm resulting in a combined statistically more relevant value with positive values indicating α-helical and negative values suggesting β-strand conformations (Fig 3A) [25]. The SSP score for each residue was calculated from ^13^C^α^ and ^13^C^β^ chemical shifts and weighted over five residues. Thereby, many residues of Nogo-A-Δ20 showed values close to zero (Fig 3A), indicating a random coil-like structure with little secondary structure elements [26–28]. However, the three segments ^561^EAIQESL^567^, ^639^EAMNVALKALGT^650^, and ^693^SNYSEIAK^700^ contained positive SSP values above 0.1 for more than five consecutive residues indicating α-helical propensity. These three stretches relate well to three α-helices that were predicted *in silico* by PSIPRED v3.3 (residues 561–566, 637–648 and 696–700; S3 Fig) [22].

In order to corroborate the proposed helical propensity of the three segments, scalar couplings ^3^*J*_HNHα_ were measured. Secondary scalar couplings, Δ^3^*J*_HNHα_, were calculated by subtracting random-coil values [29] from the experimentally measured ^3^*J*_HNHα_ data. While positive Δ^3^*J*_HNHα_ values show a tendency for β-sheets, negative values indicate turns or α-helical propensities [30]. All the three stretches proposed to be α-helical according to their secondary chemical shift values, i.e., ^561^EAIQESL^567^, ^639^EAMNVALKALGT^650^, and ^693^SNYSEIAK^700^, had negative Δ^3^*J*_HNHα_ values supporting the presence of transient α-helices in these segments (Fig 3B).

An independent measure of both disorder and secondary structure can be obtained by ^15^N{^1^H}-heteronuclear NOEs (HetNOE). While positive values close to 1 indicate structural rigidity of the backbone ^15^N-^1^H moieties, values close to 0 indicate dynamics in the range of ~1 ns, and ^15^N-^1^H moieties with negative values are highly flexible with dynamics faster than ~1 ns [31, 32]. Most of the values of Nogo-A-Δ20 were slightly positive between 0.1 and 0.2, and extended runs of positive values were especially found at the locations of all three proposed α-helical stretches (Fig 3C). In addition, the N- and C-terminal regions of Nogo-A-Δ20 showed an elevated rigidity (Fig 3C). Overall, the HetNOE data indicate a highly flexible state for Nogo-A-Δ20, as commonly found in IDPs [33, 34].

**Fig 4:**
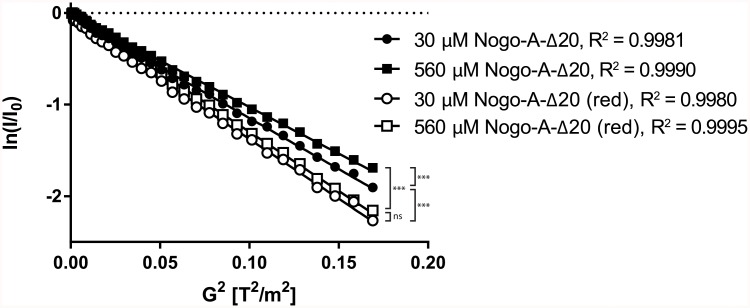
NMR diffusion experiments on Nogo-A-Δ20. Logarithmic intensities of Nogo-A-Δ20 at different conditions were plotted against its dependence on gradient strength. Nogo-A-Δ20 was measured with pulsed field gradient experiments at two concentrations (30 and 560 μM), shown as closed circles and squares, respectively. The diffusion coefficient was calculated according to [36], resulting in *D*_30 μM_ = 2.18 ± 0.02×10^-11^ m^2^/s and *D*_560 μM_ = 2.00 ± 0.01×10^-11^ m^2^/s (mean ± SD). Nogo-A-Δ20 was measured again in the presence of the reducing agent TCEP, shown as open circles (30 μM) and squares (560 μM). Here, the diffusion coefficients equalled *D*^red^_30 μM_ = 2.53 ± 0.01×10^-11^ m^2^/s and *D*^red^_560 μM_ = 2.55 ± 0.02×10^-11^ m^2^/s. ***, p < 0.0001; ns, not significant; red, reduced.

Clustering of Nogo-A-Δ20 and of full-length Nogo-A is a highly discussed topic [7, 14, 35]. Pulse-field gradient NMR spectroscopy experiments were therefore recorded to determine the diffusion coefficient *D* of Nogo-A-Δ20 at two different concentrations. *D*_30 μM_ was found to be 2.18 ± 0.02×10^-11^ m^2^/s (mean ± SD), and *D*_560 μM_ equalled 2.00 ± 0.01×10^-11^ m^2^/s (Fig 4). Since two cysteines are present in Nogo-A-Δ20, we also tested the influence of their oxidation state on the diffusion coefficient. *D*^red^_30 μM_ in the presence of the reducing agent tris (2-carboxyethyl) phosphine (TCEP) was increased to 2.53 ± 0.01×10^-11^ m^2^/s, and *D*^red^_560 μM_ to 2.55 ± 0.02×10^-11^ m^2^/s (Fig 4). These results indicate that Nogo-A-Δ20 dimerises under non-reducing conditions, although no significant chemical shift changes in 2D [^15^N, ^1^H]-HSQC NMR spectra could be observed (S4 Fig).

### Titration of S1PR2 Fragments to Nogo-A-Δ20

It has been shown that Nogo-A-Δ20 binds to isolated extracellular loops (ECL) 2 and 3 of sphingosine 1-phosphate receptor 2 (S1PR2) with affinities in the nanomolar range [12]. In order to identify the binding interface between ECL peptides and Nogo-A-Δ20 at atomic resolution, ligand titration studies were performed using NMR spectroscopy.

**Fig 5:**
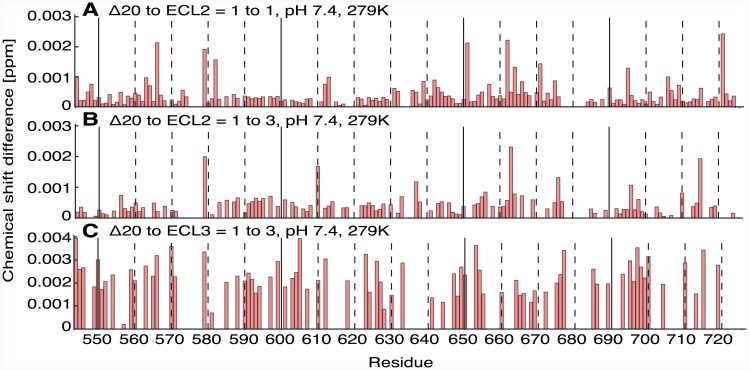
Chemical shift perturbations upon titration of ECLs of S1PR2 to Nogo-A-Δ20. Nogo-A-Δ20 chemical shift difference (CSD) of the combined ^15^N and ^1^H chemical shifts between free Nogo-A-Δ20 and Nogo-A-Δ20 in presence of ECLs. **A**: 1 to 1 ratio (ECL2) at pH 7.4 and 6°C. **B**: 1 to 3 ratio (ECL2) at pH 7.4 and 6°C. **C**: 1 to 3 ratio (ECL3) at pH 7.4 and 6°C. The chemical shift differences are smaller than 0.005 ppm indicating no chemical shift changes of Nogo-A-Δ20 protein upon ligand titration.

**Fig 6:**
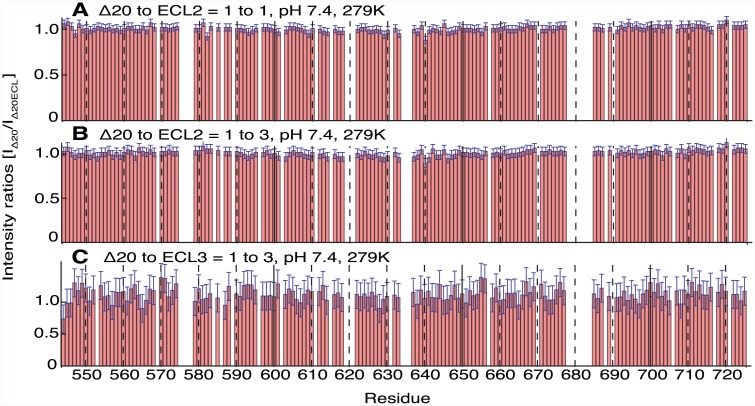
Intensity ratios between Nogo-A-Δ20 in the presence vs. absence of ECLs. **A**: 1 to 1 ratio (ECL2) at pH 7.4 and 6°C. **B**: 1 to 3 ratio (ECL2) at pH 7.4 and 6°C. **C**: 1 to 3 ratio (ECL3) at pH 7.4 and 6°C. The values are corrected for the volume decrease upon ligand titration. Minor deviations from 1 can be explained by imperfectly tuned pH and temperature. Error bars were calculated using Gaussian error propagation.

First, ECL2 was titrated to ^15^N-labeled Nogo-A-Δ20 at different molar ratios. A [^15^N, ^1^H]-HSQC spectrum with a resolution of 0.04 ppm in the ^15^N and 0.028 ppm in the ^1^H dimension was measured for each titration step at 6°C and pH 7.4 including a reference without addition of ECL2 (Fig 5 and S5 and S6 Figs). Even with a threefold excess of ECL2, no cross peak shifts were detected when compared to the spectrum without ECL2 (Fig 5B and S5C Fig). Normalized chemical shift changes of the combined ^15^N and ^1^H residues of Nogo-A-Δ20 were smaller than 0.005 ppm. These values were below the combined ^15^N and ^1^H detection resolution of 0.07 ppm, indicating the absence of conformational changes upon ECL2 addition. Since pronounced chemical shift changes were observed for several peaks of Nogo-A-Δ20 upon decreased pH (S7 Fig), it was hypothesised that a lower pH might be necessary for binding. However, a reduction of pH from 7.4 to 6.4 did not result in any peak shifts upon ECL2 titration (S6A and S5B Figs). Furthermore, a temperature increase from 6°C to 15°C to match the conditions of a previously published binding study more closely [12] did not result in any peak shifts upon ECL2 titration (S5D and S6B Figs). Subsequently, Nogo-A-Δ20 was investigated upon ECL3 titration. Similarly, no peak shifts could be detected (Fig 5C and S8A Fig). ECL3 titration also did not perturb the [^15^N, ^1^H]-HSQC spectra of Nogo-A-Δ20 in the presence of 5 mM TCEP, suggesting no dependence of binding on the oxidation state of Nogo-A-Δ20 cysteines (S6C Fig). Since the presence of zinc increases the α-helical content of Nogo-A-Δ20 [21], 4 mM zinc ions were added to the sample. Again, no changes in the spectra could be detected upon ECL3 addition, indicating that zinc ions do not facilitate ECL binding (S6D and S8B Figs). Finally, as FC12 is required for folding of Nogo-66 [19], we explored the possibility that Nogo-A-Δ20 only binds to ECL2 in the presence of FC12. However, no changes in the Nogo-A-Δ20 CD spectrum were observed when ECL2 was added in the presence of FC12 (Fig 1). The missing shifts of [^15^N,^1^H]-HSQC peaks might be explained by an intermediate exchange of the bound and unbound state. In this time regime, decreases of intensities of the amino acid residues participating in an interaction are anticipated. Therefore, the intensity ratios of Nogo-A-Δ20 in the presence vs. absence of ECL2 and ECL3 were calculated for each residue (Fig 6 and S9 Fig). The intensity ratios at pH 7.4 at 6°C were found to have a random distribution near 1 for the Nogo-A-Δ20 to ECL2 ratios of 1 to 1 and 1 to 3, indicating no intermediate exchange. Intensity ratios at pH 6.4 at 6°C and at pH 7.4 at 15°C upon addition of ECL2 and the intensity ratio at pH 7.4 at 6°C upon addition of ECL3 have a larger deviation from the value 1, which might be rather attributed to an imperfect adjustment of pH and temperature than to ECL binding.

### Isothermal Titration Calorimetry (ITC)

A third biophysical technique, ITC, was consulted to investigate the discrepancy between NMR spectroscopy and previous binding assay data [12]. Either ECL2 or ECL3 were titrated to 10 μM Nogo-A-Δ20. In both cases, only very small exothermic heat signals could be detected upon titration (S10 Fig). For ECL2, a small difference in the heat signals at low and high concentration could be observed. Furthermore, equally-sized heat signals were observed throughout the titration for ECL3.

### Cellular Activity Assay for Nogo-A-Δ20

**Fig 7:**
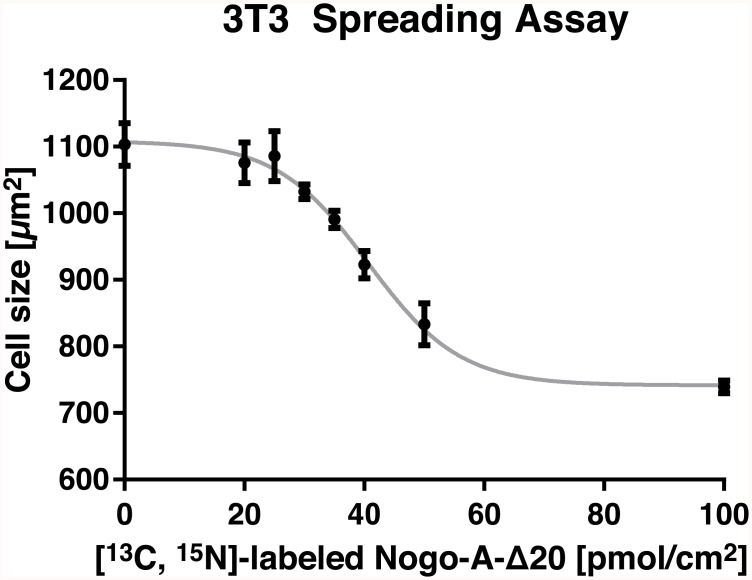
Activity assay of ^13^C, ^15^N-labelled Nogo-A-Δ20. 3T3 fibroblasts were plated on Nogo-A-Δ20 or control substrate for 1 h and fixed with paraformaldehyde. Non-linear regression reveals an IC_50_ value of ~40 pmol/cm^2^. Mean cell size ± standard deviation from three wells is shown for each concentration.

In order to confirm that the obtained structural data correspond to a biologically active protein, and in order to exclude that the lack of peak shifts upon ECL titration was due to misfolding of Nogo-A-Δ20, we performed a 3T3 fibroblast spreading assay (Fig 7). Fibroblast spreading was markedly inhibited on isotopically labelled Nogo-A-Δ20 substrate, confirming intact inhibitory activity of the protein. Importantly, the IC_50_ value was ~40 pmol/cm^2^, which is a typical potency for Nogo-A-Δ20-induced inhibition of 3T3 fibroblast spreading [37].

In summary, Nogo-A-Δ20 is an intrinsically disordered domain as indicated by CD data, [^15^N, ^1^H]-HSQC peak dispersion, secondary chemical shift analysis and dynamic studies. Within the disordered region, three contiguous segments of α-helical propensity are found. All agree well with those indicated by a computational algorithm. Diffusion coefficients of Nogo-A-Δ20 suggest a dimerisation dependent on the concentration and oxidation state of the protein. While titration of ECL2 and ECL3 to Nogo-A-Δ20 did not induce pronounced peak shifts in [^15^N,^1^H]-HSQC spectra or show marked thermal heat exchange in ITC measurements, the used batch of Nogo-A-Δ20 was found to be active in a 3T3 fibroblast spreading assay.

## Discussion

We investigated Nogo-A-Δ20 using CD and NMR spectroscopy. We were able to obtain high-quality NMR spectra for backbone assignment, which enabled us to derive structural data with atomic resolution. Our data show a high degree of disorder within the neurite growth and cell spreading inhibitory Nogo-A-Δ20 region. The largely random coil CD spectrum and narrow proton dispersion in [^15^N, ^1^H]-HSQC spectra confirm previous observations [20, 21] and are extended by the experimentally obtained secondary structure analysis of secondary chemical shifts, ^3^*J*_HNHα_ scalar coupling, as well as the high degree of flexibility indicated by HetNOE measurements. Importantly, despite the lack of fully structured regions, isotopically labelled and thrombin-cleaved Nogo-A-Δ20 exerted its typical inhibitory activity in a 3T3 fibroblast spreading assay.

Structural flexibility imposes a variety of advantages on proteins, ranging from an enlarged interaction surface and thus higher binding specificity to an elevated promiscuity towards binding partners [38–40]. As a consequence, IDPs are involved in a multitude of signalling pathways and appear in all three domains of life, i.e., Archaea, Bacteria, and Eukarya [41]. The intrinsically disordered Nogo-A-Δ20 has been shown to interact with various binding partners, such as S1PR2, tetraspanin-3, β1-integrins, cyclic nucleotide phosphodiesterase, and WWP1 [12, 13, 42–45]. In addition, clustering of N-terminal Nogo-A fragments including Nogo-A-Δ20 has been described to enhance their inhibitory potency [14, 42]. Our finding that the diffusion coefficient of Nogo-A-Δ20 is reduced at higher concentrations supports the notion of dimer formation. A high degree of flexibility might represent an important structural feature of this domain, increasing its surface area available for binding molecular target proteins and for homodimerisation. Additionally, Nogo-A is a multifaceted player implicated in neurite outgrowth inhibition, CNS development, synaptic plasticity, ER membrane morphology, and several other processes by interactions with several binding partners and multisubunit receptors [10, 46, 47]. Structural disorder could therefore allow different sets of interacting molecules to bind to the same sites within Nogo-A-Δ20 depending on the context, a model referred to as functional moonlighting [48].

Although no fully structured regions were found, we identified three segments within Nogo-A-Δ20 that appear to form transient and dynamical α-helical structures: ^561^EAIQESL^567^, ^639^EAMNVALKALGT^650^, and ^693^SNYSEIAK^700^. All of these regions seem to be α-helical based on their positive SSP and negative Δ^3^*J*_HNHα_ values, and they exhibit decreased flexibility as determined by HetNOE. Similar results were obtained for the very C-terminal four residues of Nogo-A-Δ20 (residues 722–725), which might represent the beginning of another α-helix in full-length Nogo-A. At the N-terminal boundary of Nogo-A-Δ20 (residues 548–560), flexibility is also reduced. However, the secondary structure of this region could not be determined unambiguously by SSP and Δ^3^*J*_HNHα_ evaluation.

Significant residual secondary structures are commonly found in IDPs, and they often resemble structural characteristics present in the bound state [49–53]. It has therefore been suggested that these residual structures are involved in initial molecular recognition [51, 52]. One could speculate that the α-helical structures found in Nogo-A-Δ20 also serve as such recognition sparks, forming initial contact with binding partners. Adjacent unstructured regions could then confer higher specificity to the interaction. Strikingly, ^561^EAIQESL^567^ is located in direct juxtaposition with one of the most conserved domains of Nogo-A-Δ20 (residues 554–559) that harbours a β1-integrin binding motif (S1 Fig) [43]. Similarly, ^639^EAMNVALKALGT^650^ partially overlaps with the binding epitope for the Nogo-A-neutralizing antibody 11C7 (residues 630–640) that has been shown to enhance recovery from spinal cord injury in rats and macaques [54, 55].

Forced dimerisation of Nogo-A-Δ20 has been reported to enhance the inhibitory properties of the protein [7, 14]. The markedly elevated diffusion coefficients in the presence of TCEP support dimerisation through the formation of disulphide bridges of Nogo-A-Δ20 in a non-reducing environment. Dimerisation seems to be concentration dependent, as the diffusion coefficient of Nogo-A-Δ20 in the absence of TCEP decreased at higher concentrations of Nogo-A-Δ20. A larger fragment of human Nogo-A that contained Nogo-A-Δ20 has been shown to co-exist in two disulphide isomers by non-reducing SDS/PAGE [35]. Electrophoretic mobility was enhanced in each of these disulphide isomers as compared to the reduced fragment, indicating that both isomers harbour intra-molecular disulphide bridges. By mutational analysis, the authors were able to identify four conserved cysteines that are involved in this process, though the exact connectivity remains elusive [35]. Of these conserved cysteines, only one (corresponding to rat Cys574) is located within Nogo-A-Δ20, indicating a possible intra-molecular disulphide bond with a cysteine outside of Nogo-A-Δ20. In the isolated Nogo-A-Δ20 fragment, this unpaired cysteine could contribute to non-physiological inter-molecular disulphide bridges resulting in dimerisation. Alternatively, it is possible that the second cysteine within Nogo-A-Δ20, Cys676, accounts for this observation, though it seems dispensable for overall folding [35].

Oxidation state-dependent dimerisation could be of physiological relevance for Nogo-A signalling. In order to impose its inhibitory effect on passing neurons, Nogo-A-Δ20 is presented on the surface of oligodendrocytes, where it is exposed to the oxidising environment of the extracellular space [5, 7]. On the other hand, a different membrane topology is found at the ER, where Nogo-A-Δ20 faces the reducing milieu of the cytosol [7, 11]. It can therefore be speculated that the oxidation state of Nogo-A-Δ20 at different cellular compartments contributes to its diverse functions, possibly through differential disulphide bonding and/or dimerisation. The connectivity of disulphide bridges in Nogo-A, as well as their individual contributions to folding, dimerisation, S1PR2 binding and inhibitory activity, will be the subject of future systematic mutational studies.

No chemical shift perturbations could be observed upon titration of ECL peptides, and only very small exothermal signals were detected with ITC suggesting no specific high affinity binding. This is surprising, as ECL2 and ECL3 had been shown to bind Nogo-A-Δ20 with K_D_ values of ~280 nM and ~350 nM, respectively, by microscale thermophoresis [12]. Microscale thermophoresis determines the diffusion coefficient of a labelled molecule as a function of the concentration of its binding partner. The diffusion coefficient is susceptible to various parameters such as buffer composition or size, charge, hydration shell or conformation of a molecule [56]. Therefore, not only molecular interactions are measured via microscale thermophoresis, but also conformational alterations or charge variations caused by slight changes in the buffer conditions within the titration experiment such as pH, which do not have to be induced by ligand binding. On the other hand, it should be noted that isolated ECLs are likely to assume different conformations than in the context of the whole GPCR. The physiologically relevant structure presumably depends on the relative positions of adjacent hydrophobic regions. Likewise, Nogo-A-Δ20 only represents a fragment of Nogo-A that might not include all structural features required for a physiological interaction. Future structural investigations should therefore concentrate on the full-length Nogo-A and S1PR2 proteins.

In conclusion, we have shown that biologically active Nogo-A-Δ20, while unstructured in the majority of its sequence, contains three stretches with α-helical propensity. Whereas α-helices could be involved in initial recognition and presentation of disordered regions, structural flexibility of Nogo-A-Δ20 might be essential for specific interactions with the binding partners in cellular membranes, neuritic growth cones, at CNS synapses, and in the ER. We provide further evidence that dimerisation occurs in Nogo-A-Δ20, the physiological relevance of which needs to be further investigated. However, we could not detect structural changes of Nogo-A-Δ20 upon titration of isolated ECL2 or ECL3 by NMR spectroscopy, and only minor thermal heat exchanges were observed by ITC. It will be fascinating to gain more insight on the structural basis of this clinically highly relevant signalling node.

## Material and Methods

### Expression of Isotopically Labelled Nogo-A-Δ20

Rat Nogo-A-Δ20 (residues 544–725) was cloned into the pET28 vector containing a His_6_-tag at each terminus and a T7-tag between the N-terminal His_6_-tag and Nogo-A-Δ20 [7]. ^15^N- or ^13^C,^15^N-labelled Nogo-A-Δ20 was expressed in One Shot BL21 (DE3) strain of *E*. *coli* in M9 minimal medium with max. 4 g/L D—glucose-^13^C_6_ (^13^C > 99%) or 8 g/L D-glucose-^12^C_6_ and 1 g/L ^15^N-ammonium chloride (^15^NH_4_Cl, ^15^N > 98%) purchased from Sigma-Aldrich (Buchs, Switzerland). Bacteria were grown at 37°C at 100 rpm until the OD_590_ reached 1.2, transferred to 30°C and induced with 1 mM IPTG. The fusion protein was expressed for 8 hours and cells were harvested by centrifugation. The wet pellet was stored at -80°C.

### Purification of Nogo-A-Δ20

All of the following purification steps were performed at 4°C. A frozen pellet of 1 L of bacterial culture was thawed on ice and resuspended in 50 mL lysis buffer (20 mM NaH_2_PO_4_, 500 mM NaCl, 20 mM imidazole, pH 7.4). 0.5 mg/mL lysozyme, 0.5 mM PMSF, and 1 protease inhibitory tablet (Roche Diagnostics GmbH, Mannheim, Germany) were added. The lysate was stirred for 20 min. Cells were further disrupted by passing twice through a 110S microfluidizer (Microfluidics, Newton, Massachusetts, USA) at 40 PSI. The suspension was centrifuged at 40'000 rpm (125171 g) for 30 min (Optima L-90K Ultracentrifuge, rotor Ti-45, Beckman Coulter International, S.A., Nyon, Switzerland) to pellet cellular debris [7]. The supernatant of the centrifugation was bound to 3 mL Ni-NTA Agarose from Qiagen (Merck KGaA, Darmstadt, Germany) via batch mode during 2 h. The Ni-NTA was washed with 30 mL lysis buffer, eluted with ca. 5 mL elution buffer (20 mM NaH_2_PO_4_, 500 mM NaCl, 500 mM imidazole, pH 7.4) via gravity flow and collected in 0.5 mL fractions. The elution buffer was exchanged to PBS buffer with a pre-packed and disposable PD-10 desalting column (GE Healthcare Life Sciences, Buckinghamshire, UK). To remove the N-terminal His_6_-tag, bovine thrombin (Sigma-Aldrich, Buchs, Switzerland) was added to the desalted sample with the ratio of 2 NIH units of thrombin per ca. 1 mg desalted Nogo-A-Δ20 for 1 hour. The cleaved fusion protein was purified on a Highload^™^ 26/60, Superdex^™^ 75 column using an Äkta FPLC system (prep grade, GE Healthcare, Uppsala, Sweden).

To exclude batch-to-batch variations, 6 L of ^15^N-labelled Nogo-A-Δ20 were expressed, purified, shock frozen in aliquots, and finally stored at -80°C until usage for ECL titration.

### CD spectroscopy

CD measurements were carried out on a Jasco J815. The spectra were scanned from 260–198 nm at 20 nm/min with 1 nm band-pass, 4 seconds integration and averaged over 2 repetitions. The measurements of Nogo-A-Δ20 were executed in PBS at 25°C with a concentration of 10 μM. Dodecylphosphocholine (FC12; Affymetrix, Santa Clara, CA, USA) and ECL2 (see NMR section) were added to final concentrations of 6.67 mM and 10 μM, respectively.

### NMR spectroscopy

The concentration of isotopically labelled Nogo-A-Δ20 for the NMR measurements was between 80–400 μM in PBS buffer containing 95% H_2_O and 5% D_2_O at pH 7.4. The experiments were recorded on 600 MHz, 700 MHz, or 900 MHz Bruker NMR spectrometers (Bruker BioSpin AG, Fällanden, Switzerland) equipped with either TCI or TXI cryoprobes. For the amino acid sequence assignment, a [^15^N, ^1^H]-HSQC and a set of four triple-resonance experiments were measured at 6°C. The chemical shifts of the amide proton, the amide nitrogen, the ^13^C^α^ and ^13^C^β^ were obtained using the triple-resonance experiments HNCA (80[F3] × 80[F2] × 1024[F1] complex data points, 16 number of scans, 0.8 s relaxation delay, WATERGATE for water suppression and gradient pulses) [57] and HNCACB (100[F3] × 80[F2] × 1024[F1] complex data points, 32 number of scans, 1 ms relaxation delay, preservation of equivalent path (PEP) sensitivity enhancement and gradient pulses) [58]. Additionally, an HNN spectrum was recorded connecting N_i_ to N_i-1_ and N_i+1_ (1024[F3] × 144[F2] × 256[F1] complex data points, 16 number of scans, 1 s relaxation delay with gradient enhancement) [59]. To correlate ^1^H^α^ to ^13^C^α^ and to N_i_ and N_i-1_, a HCAN spectrum (124[F3] × 114[F2] × 1024[F1] complex data points, 16 number of scans, 1 s relaxation delay) using PEP was recorded [23], enabling assignment through proline residues.

The difference of the chemical shifts of the measured δ^13^C^α^ and δ^13^C^β^ and random coil values [60] were calculated for the secondary chemical shift analysis. Composite values of Δδ^13^C^α^ and ΔδC^β^ were calculated using the SSP algorithm from Forman-Kay group [25]. The algorithm combines individual contributions of chemical shifts regarding their sensitivity to α- and β-structure from different nuclei into a score. Hereby, the observed chemical shift differences of a residue are weighted against the expected chemical shift differences for a secondary structure. To minimize contributions from chemical shifts that are poor measures of secondary structures, e.g., glycines, the algorithm additionally averages the score over five residues. The final score of a residue ranges from 1 to -1, indicating fully formed α-helical or β-strand conformations, respectively.

An intensity modulated [^15^N, ^1^H]-HSQC [61] was measured to obtain the ^3^*J*_HNHα_ scalar couplings (16 number of scans, 1 s relaxation delay, 2 τ = time for evolution of ^3^*J*_HNHα_: 18 ms). The intensity ratios of the relation I_m_/I_d_ = cos(π(^3^J_HNHα_)2 τ) were used for the calculation of the coupling constant ^3^*J*_HNHα_, I_m_ being the intensity of the modulated spectra and I_d_ that of decoupled ones. The experimentally obtained ^3^*J*_HNHα_ was multiplied by a correction coefficient of the magnitude of 1.06 due to the different relaxation properties of the in- and antiphase magnetisation of the H^N^ compared to the H^α^ [61]. The secondary scalar couplings, Δ^3^*J*_HNHα_, were calculated by subtracting the corresponding random-coil values [29] from the experimentally measured ^3^*J*_HNHα_ data.

Dynamics of Nogo-A-Δ20 were examined with a ^15^N{^1^H}-HetNOE experiment (8 number of scans, 6 s relaxation delay) [32]. The HetNOE was estimated by dividing I_S_, the intensity of the saturated spectrum, by I_U_, the intensity of the corresponding peak in the unsaturated spectrum. Error bars for the HetNOE plot were calculated using Gaussian error propagation [62].

To determine the diffusion coefficient of reduced and unreduced Nogo-A-Δ20, pulsed field gradient experiments [36] were measured (30[F2] × 16384[F1] complex data points, gradient between 5–75%, Δ of 200 ms, δ of 5.5 ms, 32 or 128 number of scans, 10 s relaxation delay, WATERGATE for water suppression). The sample constituted of either 560 μM in absence or presence of 2 mM tris(2-carboxylethyl)phosphine hydrochloride (TCEP, Sigma-Aldrich, Buchs, Switzerland) or 30 μM in absence or presence of 5 mM TCEP at pH 7.4 and 6°C. The experimental data points were fitted according to [36]. Gradient strength was calibrated with H_2_O, for which coefficient is known [63, 64]. Linear regression and an statistical analysis of the slope differences were performed in Prism 5 (GraphPad Software, La Jolla, CA, USA), which follows a calculation method that is equivalent to ANCOVA [65].

For the ECL titration, [^15^N,^1^H]-HSQC experiments were measured with a resolution of at least 300[F2] × 2048[F1] with a maximal evolution time of 82 ms and 114 ms for ^15^N and ^1^H frequencies, yielding a resolution of 0.04 ppm and 0.028 ppm, respectively. The combined ^15^N and ^1^H resolution was 0.07 ppm. ECL2 (peptide sequence NCLNQLEACSTVLPLYAKHYVL) and ECL3 (SILLLDSTCPVRACPVLYK) were purchased from JPT Peptide Technologies GmbH (Berlin, Germany). The concentration used for ^15^N-labeled Nogo-A-Δ20 was 30 μM, 88 μM or 120 μM and the following molar ratios were measured: Nogo-A-Δ20: ECL2: 1: 1 and 1: 3, Nogo-A-Δ20: ECL3: 1: 3. The sample for ECL2 titration was measured at different pH values (pH 7.4 and 6.4) and different temperatures (6°C and 15°C). The Nogo-A-Δ20 sample for the ECL3 titration was measured at pH 7.4 at 6°C in the presence or absence of either 5 mM TCEP or 4 mM ZnCl_2_. The chemical shift differences (CSD) between the peaks of Nogo-A-Δ20 alone and those in presence of an ECL in the [^15^N, ^1^H]-HSQC were calculated using the following equation [66]:

CSD=0.5×[(H1A−H1T)2+0.14×(N15A−N15T)2]

where ^1^H_A_ and ^1^H_T_ are the ^1^H chemical shifts of Nogo-A-Δ20 alone and in the presence of ECL, and ^15^N_A_ and ^15^N_T_ are the ^15^N chemical shifts of Nogo-A-Δ20 alone and in the presence of ECL. Additionally, the combined resolution of ^1^H and ^15^N was calculated with this equation by inserting the ^1^H and ^15^N resolution instead of the chemical shift difference for ^1^H and ^15^N, respectively. The spectra were processed with Topspin 3.1 (Bruker) before analysis. The amino acid residue assignment was accomplished using the CcpNmr software [67].

### Isothermal Titration Calorimetry (ITC)

Nogo-A-Δ20, as well as ECL2 and ECL3 of S1PR2 were dialysed against PBS (pH 7.4) at 4°C overnight. ITC experiments were carried out at 25°C on a MicroCal VP-ITC instrument (Malvern Instruments, Worcestershire, UK) with a cell volume of 1400 μL and a syringe volume of 300 μL. Each experiment consisted of an initial injection of 2 μL, followed by 29 injections of 10 μL. Stirring speed was 300 rpm. Nogo-A-Δ20 was 10 μM in the cell, whereas ECL peptides were 150 μM in the syringe. All data were analysed with the Origin software supplied by the manufacturer.

### 3T3 Fibroblast Spreading Assay

Four-well plates (Greiner BioOne GmbH, Frickenhausen, Germany) were coated overnight at 4°C with a dilution series of [^13^C, ^15^N]-labelled Nogo-A-Δ20 in PBS, ranging from 0 to 100 pmol per cm^2^ growth area. The next day, wells were washed three times with PBS. NIH 3T3 fibroblasts (ATCC, Wesel, Germany) were briefly trypsinised, plated on Nogo-A-Δ20 or plastic control substrate at 7’000 cells per cm^2^, and incubated for 1 h at 37°C and 5% CO_2_. Cells were fixed with warm 4% paraformaldehyde (Sigma-Aldrich, Buchs, Switzerland) in PBS for 20 min at RT, and washed three times with PBS at RT. Permeabilisation/blocking buffer [2% normal goat serum (Jackson Laboratories, ME, USA), 0.2% Triton-X100 (AppliChem, Darmstadt, Germany), 0.004% fish skin gelatine (Sigma-Aldrich, Buchs, Switzerland) in PBS at pH 7.4] was added for permeabilisation at 4°C overnight. Cells were then incubated with DAPI (1:1000, Life Technologies, Carlsbad, CA, USA) and Alexa Fluor 488-labelled phalloidin (1:100, Life Technologies, Carlsbad, CA, USA) in permeabilisation/blocking buffer for 1 h at RT to stain nuclei and the actin cytoskeleton, respectively. Finally, cells were washed three times with PBS and coverslipped in fluorescence mounting medium (Dako Schweiz AG, Baar, Switzerland). An Axioskop 2 mot plus fluorescence microscope (Carl Zeiss AG, Feldbach, Switzerland) was used for automatic acquisition of DAPI and phalloidin images for 28 positions in each well. CellProfiler software was employed to measure the sizes of only non-clumped cells [68]. Finally, non-linear regression was performed in Prism 5 (GraphPad Software, La Jolla, CA, USA).

## Supporting Information

S1 Fig Sequential backbone assignment of Nogo-A-Δ20. Assigned residues are designated in black, unassigned ones in grey. Bold type indicates the Nogo-A-Δ20 segment; the T7-tag and the His_6_-tag are indicated in lowercase. 94% of the non-proline δC^α^ frequencies within the 182 amino acid residues of Nogo-A-Δ20 were assigned. Residues with transient α-helical conformations according to the combined secondary chemical shift values are highlighted in yellow. Amino acid residues are numbered as found in the rat Nogo-A protein.
(EPS)

S2 Fig Secondary chemical shift analysis. Δδ^13^C^α^ and Δδ^13^C^β^ are shown individually in red and blue. Stretches of positive Δδ^13^C^α^ and negative Δδ^13^C^β^, indicating α-helical propensity, are found at residues ^561^EAIQESL^567^, ^639^EAMNVALKALGT^650^, and ^693^SNYSEIAK^700^.
(EPS)

S3 Fig PSIPRED v3.3 secondary structure prediction for Nogo-A-Δ20. Several α-helical domains and two β-strands are predicted with different confidence scores. Especially the α-helices around amino acid residues 563, 643 and 697 have elevated likeliness to occur.
(EPS)

S4 Fig [^15^N, ^1^H]-HSQC spectra of 560 μM Nogo-A-Δ20 alone and in the presence of 2 mM TCEP at pH 7.4 and 6°C. Spectrum of Nogo-A-Δ20 alone is shown in red contours, while the spectrum upon addition of TCEP is in blue. The spectra revealed no marked chemical shift differences in the presence and absence of TCEP.
(EPS)

S5 Fig [^15^N, ^1^H]-HSQC spectra of 88 μM Nogo-A-Δ20 alone and in the presence of ECL2 at different pH values and temperatures. For each subfigure, the spectrum of Nogo-A-Δ20 alone is shown in red contours, while the spectrum upon addition of an ECL fragment is colour coded in blue. **A**: Nogo-A-Δ20 to ECL2 ratio of 1 to 1 at pH 7.4 and 6°C. **B**: Nogo-A-Δ20 to ECL2 ratio of 1 to 1 at pH 6.4 and 6°C. **C**: Nogo-A-Δ20 to ECL2 ratio of 1 to 3 at pH 7.4 and 6°C. **D**: Nogo-A-Δ20 to ECL2 ratio of 1 to 3 at pH 7.4 and 15°C. No significant peak shifts occurred upon ECL2 titration.
(EPS)

S6 Fig Chemical shift perturbations upon titration of ECLs of S1PR2 to Nogo-A-Δ20. Nogo-A-Δ20 chemical shift difference (CSD) of the combined ^15^N and ^1^H chemical shifts between free Nogo-A-Δ20 and Nogo-A-Δ20 in presence of ECLs. **A**: 1 to 1 ratio (ECL2) at pH 6.4 and 6°C. **B**: 1 to 3 ratio (ECL2) at pH 7.4 and 15°C. **C**: 1 to 3 ratio (ECL3) at pH 7.4 and 6°C in the presence of 5 mM TCEP. **D**: 1 to 3 ratio (ECL3) at pH 7.4 and 6°C in the presence of 4 mM zinc ions. The chemical shift differences are smaller than 0.005 ppm indicating no chemical shift changes of Nogo-A-Δ20 protein upon ligand titration.
(EPS)

S7 Fig [^15^N, ^1^H]-HSQC of Nogo-A-Δ20 at pH 7.4 and 6.4 at 6°C. Red spectrum corresponds to Nogo-A-Δ20 at pH 7.4 and the green spectrum at pH 6.4. When comparing the spectra at pH 7.4 and 6.4, pronounced chemical shifts are observable.
(EPS)

S8 Fig [^15^N, ^1^H]-HSQC of 120 μM Nogo-A-Δ20 alone and in the presence of ECL3 at pH 7.4 and 6°C. **A**: Nogo-A-Δ20 to ECL3 ratio of 1 to 3 at pH 7.4 and 6°C. **B**: Nogo-A-Δ20 to ECL3 ratio of 1 to 3 at pH 7.4 and 6°C in the presence of 4 mM zinc ions. Pink and green spectrum: Nogo-A-Δ20 alone and in the presence of zinc, respectively; blue and purple spectrum: Nogo-A-Δ20 in the presence of ECL3 and additionally zinc. No significant peak shifts occurred upon ECL3 titration in the presence or absence of zinc ions.
(EPS)

S9 Fig Intensity ratios between Nogo-A-Δ20 in the presence vs. absence of ECLs. **A**: 1 to 1 ratio (ECL2) at pH 6.4 and 6°C. **B**: 1 to 3 ratio (ECL2) at pH 7.4 and 15°C. **C**: 1 to 3 ratio (ECL3) at pH 7.4 and 6°C in the presence of 5 mM TCEP. The values are corrected for the volume decrease upon ligand titration. Minor deviations from 1 can be explained by imperfectly tuned pH and temperature. Error bars were calculated using Gaussian error propagation.
(EPS)

S10 Fig ITC curves. Plots showing titration of ECLs into Nogo-A-Δ20. **A**: ECL2 titration into Nogo-A-Δ20 showing only very small thermal heat exchanges. **B**: ECL3 addition to Nogo-A-Δ20 shows only equally-sized heat signals throughout titration.
(EPS)
